# Systemic Bacillus Calmette-Guérin (BCG) Infection in a Patient With Non-Muscle-Invasive Bladder Cancer: A Case Report

**DOI:** 10.7759/cureus.77558

**Published:** 2025-01-16

**Authors:** Ana S Ramoa Oliveira, Raquel Afonso, Eduardo Macedo, Bernardo Silvério, Ana Oliveira

**Affiliations:** 1 Internal Medicine, Hospital de Braga, Braga, PRT; 2 Internal Medicine, Centro Hospitalar do Médio Ave, Vila Nova de Famalicão, PRT

**Keywords:** bcg instillation, bcg therapy, bladder cancer, multiorgan dysfunction, non-muscle-invasive bladder cancer, systemic bcg infection

## Abstract

Intravesical Bacillus Calmette-Guérin (BCG) instillation is a widely used adjuvant therapy for non-muscle-invasive bladder cancer (NMIBC), typically associated with a favorable safety profile. However, severe complications such as systemic BCG infection, though rare, can occur and are potentially life-threatening. Diagnosing systemic BCG infection is challenging due to frequently negative microbiological results. We report the case of a 67-year-old immunocompetent man who developed a systemic BCG infection after undergoing BCG therapy for NMIBC. Initially misdiagnosed as a urinary tract infection, his condition worsened despite antibiotic treatment. The diagnosis of BCG sepsis was suspected after prolonged fever, asthenia, and other systemic symptoms. Prompt initiation of antituberculosis treatment with rifampicin, ethambutol, and isoniazid led to a full recovery. This case underscores the critical importance of early recognition and treatment of systemic BCG infections, particularly in patients presenting with persistent fever and no clear source of infection.

## Introduction

Intravesical Bacillus Calmette-Guérin (BCG) therapy, utilizing a live attenuated strain of *Mycobacterium bovis*, is the most effective treatment for high- or intermediate-risk non-muscle-invasive bladder cancer (NMIBC) [1-3]. Administered via urethral catheterization, BCG reduces the risk of recurrence and progression, improving long-term survival [1,2]. Though generally well tolerated, BCG can cause local and systemic complications [3-7]. Acute symptoms like fever, dysuria, and mild hematuria are common, typically resolving within 48 hours [3]. However, severe complications, including granulomatous prostatitis, pneumonitis, and sepsis, are rare but can be life-threatening [5-7].

Systemic BCG infection, although uncommon, may arise when the attenuated bacteria disseminate beyond the bladder [3-6]. It often presents with nonspecific symptoms such as fever, weight loss, and night sweats, making diagnosis challenging [3-6]. Without prompt treatment, it can lead to severe outcomes [3-6].

This case underscores the importance of vigilance in patients undergoing BCG therapy, particularly when symptoms persist despite standard antibiotic treatment. Early recognition and intervention are crucial to prevent severe outcomes associated with systemic BCG infections.

## Case presentation

A 67-year-old man with a history of hypertension, dyslipidemia, type 2 diabetes, and heavy smoking (50 pack-year) underwent transurethral resection of the bladder. Histological analysis revealed high-grade papillary urothelial carcinoma. Imaging studies confirmed the absence of locoregional or metastatic disease. He was prescribed a three-year adjuvant schedule of intravesical immunotherapy with BCG instillations, consisting of a six-week induction course (once weekly), followed by maintenance doses (once weekly for three weeks) at three, six, 12, 18, 24, 30, and 36 months. Follow-up cystoscopies were scheduled every three months.

The initial treatments were uneventful. However, 12 months into therapy, two days after the 14th treatment, he developed macroscopic hematuria, suprapubic pain, and fever. Major complications were ruled out, and he was discharged with a course of amoxicillin/clavulanic acid (875 mg/125 mg every 12 hours). Despite treatment, his symptoms persisted, prompting a return to the emergency department.

A computed tomography (CT) scan of the thorax, abdomen, and pelvis excluded significant complications, and he was discharged with a second antibiotic, considering a possible urinary tract infection (ciprofloxacin 500 mg every 12 hours). When symptoms continued unabated, he was hospitalized and treated with a seven-day course of intravenous ceftriaxone (2 g once daily) for a possible urinary infection. Urine cultures remained consistently negative throughout.

Despite multiple courses of antibiotics, the patient’s symptoms persisted, warranting further evaluation. Further questioning revealed significant weight loss over the previous month, worsening asthenia, occasional fever spikes (maximum 38.9ºC), and night sweats.

**Figure 1:**
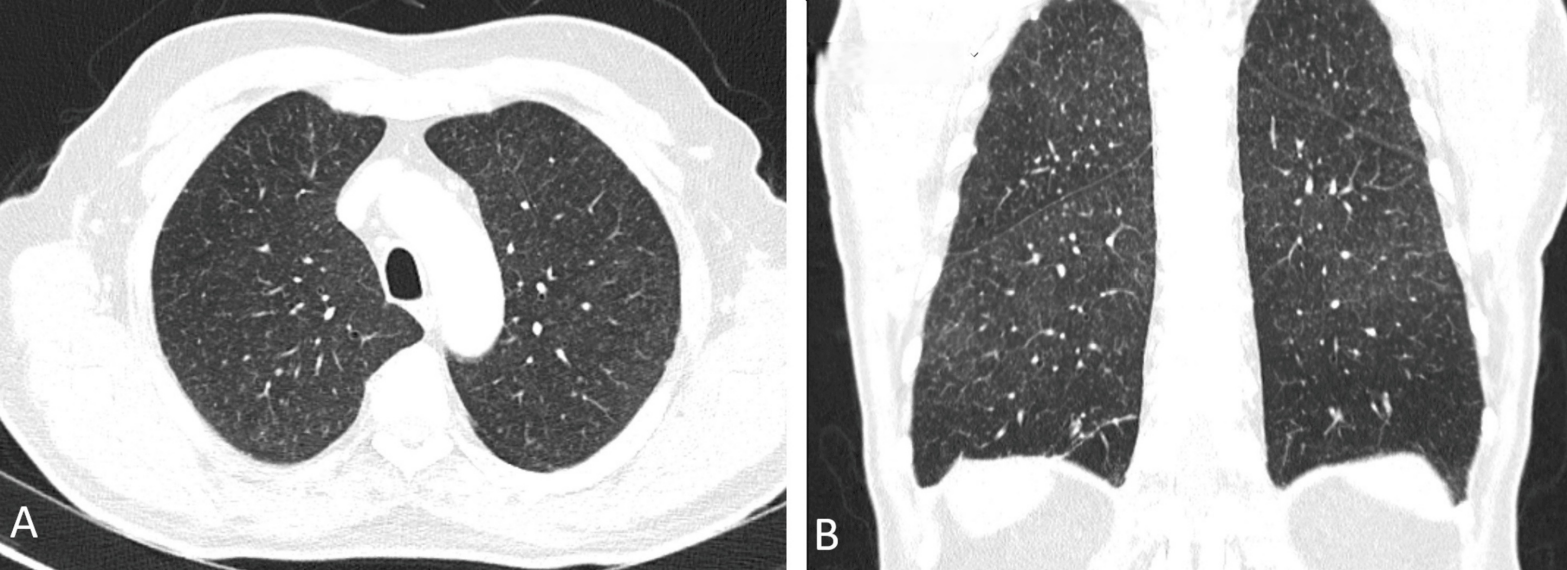
Computed tomography. Axial (A) and coronal (B) images showing expanded lungs with peribronchovascular interstitial thickening of diffuse micronodular appearance in both lungs, suggestive of a nonspecific inflammatory process of the small airways. No pulmonary consolidations are present.

On physical examination, he presented with hypotension (BP: 90/35 mmHg), tachycardia (HR: 120 bpm), and inspiratory crackles on pulmonary auscultation. Arterial blood gas analysis showed hypoxemia with respiratory alkalosis (Table 1). Thoracic CT imaging showed "diffuse micronodular peribronchovascular interstitial thickening in both lungs, suggestive of a nonspecific inflammatory process" (Figure 1). Laboratory tests (Table 1) indicated hepatitis without cholestasis, as well as acute kidney injury. Multiorgan dysfunction was evident, with cardiovascular, hepatic, renal, and pulmonary involvement.

**Table 1:** Laboratory results paO_2_, partial pressure of oxygen; pCO_2_, partial pressure of carbon dioxide; HCV, hepatitis C virus; VDRL, Venereal Disease Research Laboratory.

Parameter	Patient's Value	Normal Range
PaO_2_ (arterial oxygen) on admission	50 mmHg	75-100 mmHg
pH (arterial blood gas) on admission	7.51	7.35-7.45
pCO_2_ (arterial blood gas) on admission	30 mmHg	35-45 mmHg
Hemoglobin on admission	14.4 g/dL	13.5-17.0 g/dL
Leukocytes on admission	5.100 uL	4.0-11.0 uL
Aspartate aminotransferase on admission	78 U/L	10-40 U/L
Alanine aminotransferase on admission	73 U/L	7-56 U/L
Gamma-glutamyl transferase on admission	50 U/L	9-48 U/L
Alkaline phosphatase on admission	83 U/L	44-121 U/L
Total bilirubin on admission	0.7 mg/dL	0.1-1.2 mg/dL
Direct bilirubin on admission	0.2 mg/dL	0-0.3 mg/dL
Lactate dehydrogenase on admission	318 U/L	120-246 U/L
Urea on admission	47 mg/dL	19-49 mg/dL
Creatinine on admission	1.7 mg/dL	0.6-1.2 mg/dL
C-reactive protein on admission	105.3 mg/L	<5 mg/L
Anti-HIV antibodies	Negative	-
Hepatitis B surface antigen	Negative	-
Anti-hepatitis B surface antibody	Negative	-
Anti-hepatitis B core antibody	Negative	-
Anti-HCV antibodies	Negative	-
VDRL	Negative	-
Antinuclear antibodies	Negative	-
Anti-dsDNA antibodies	Negative	-
Anti-LKM-1 antibodies	Negative	-
Anti-liver cytosol antibodies	Negative	-
Anti-soluble liver antigen	Negative	-
Anti-mitochondrial antibodies	Negative	-
Rheumatoid factor	Negative	-
Anti-cyclic citrullinated peptide antibodies	Negative	-
Anti-neutrophil cytoplasmic antibodies	Negative	-
Anti-basement membrane antibodies	Negative	-
IgG	1200 mg/dL	700-1600 mg/dL
IgM	80 mg/dL	40-230 mg/dL
IgA	100 mg/dL	70-400 mg/dL
C3	121 mg/dL	90-180 mg/dL
C4	35 mg/dL	10-40 mg/dL
Blood cultures	Negative	-
Urine mycobacteriological analysis	Negative	-
Sputum mycobacteriological analysis	Negative	-
Sputum microbiological analysis	Negative	-
Urine microbiological analysis	Negative	-
Detection of mycobacteria by polymerase chain reaction	Negative	-

Despite extensive evaluation, including serological and immunological tests, all markers were negative, and cultures failed to identify an infectious agent. Based on the clinical findings and the patient’s history, a diagnosis of systemic BCG infection was strongly suspected.

Given the temporal association with BCG instillation and the presence of multiorgan dysfunction, a presumptive diagnosis of BCG sepsis was made. Antituberculosis therapy was initiated with rifampicin (600 mg once daily), ethambutol (1200 mg once daily), and isoniazid (300 mg once daily). The patient showed rapid improvement and was discharged after 23 days of hospitalization. Antituberculosis therapy was continued for six months without side effects, and BCG immunotherapy was permanently discontinued. There was no evidence of infection recurrence or cancer at the one-year follow-up.

## Discussion

Intravesical instillation of BCG is a widely effective adjuvant therapy for NMIBC [1,2]. It is generally well tolerated, with limited and self-limiting adverse effects [1,2,7]. However, severe complications, such as systemic BCG infection, are rare but life-threatening [5-7]. Despite these complexities, BCG immunotherapy remains the most effective treatment for NMIBC [1,2].

The exact mechanism of systemic BCG infection remains unclear, with proposed hypotheses including *Mycobacterium bovis* dissemination and immune hypersensitivity reactions [3,8]. Histopathological and clinical distinctions between infection and hypersensitivity remain difficult in many cases [8].

Disseminated BCG infection, although rare, represents a potentially fatal complication of BCG therapy [3,5-7]. It is not always preceded by visible traumatic catheterization, although this is a known risk factor [3,9]. Adverse effects from intravesical BCG instillation can manifest immediately or even years after the procedure [7,9].

BCG infection typically presents with persistent fever, weight loss, and night sweats, often weeks to months after therapy [3]. Symptoms may mimic tuberculosis or other systemic infections. Diagnosis relies heavily on clinical suspicion, as the sensitivity of acid-fast bacillus smear and mycobacterial culture is limited [3,5,10,11]. The development of a new, highly sensitive, and specific real-time PCR assay can improve the detection of *Mycobacterium tuberculosis* complex isolates in specimens [11,12]. However, in our case, as in other reviewed cases, the PCR test yielded negative results [3]. Therefore, in cases with high clinical suspicion, treatment should be initiated promptly, as was done for our patient, without waiting for laboratory confirmation.

When evaluating the risk of systemic infection, it is crucial to recognize that BCG instillation immediately following transurethral resection of a bladder tumor or traumatic catheterization is an absolute contraindication [9]. Furthermore, BCG therapy should be postponed in patients presenting with fever or suspected urinary tract infections until these symptoms resolve [9]. Routine pre-procedure urinalysis is recommended to enhance patient safety, even in asymptomatic individuals [9]. While immunosuppression, such as in HIV-positive patients, is frequently considered a relative contraindication to BCG therapy, studies by Herr and Dalbagni have demonstrated that BCG immunotherapy remains both effective and safe for immunocompromised cancer patients [13].

This case highlights a disseminated BCG infection evolving with multiorgan dysfunction due to delays in diagnosis. It is important to emphasize the delay in diagnosis and the potential dangers of not suspecting systemic BCG infection early, especially when symptoms are misattributed to more common conditions like a urinary tract infection, leading the patient to undergo multiple cycles of antibiotic therapy unnecessarily. We use this case to highlight to all general clinicians that a high index of suspicion should be raised for systemic dissemination in patients presenting with persistent fever, accompanied by fatigue and night sweats, who are currently under BCG treatment or have undergone it in the past.

Treatment typically involves a combination of antituberculosis medications, although the optimal duration of therapy has not yet been definitively established [8]. In cases of multisystemic involvement, corticosteroid therapy should also be considered, although no standardized regimen has been recommended [3].

## Conclusions

Disseminated BCG infection is a rare but serious complication of intravesical BCG therapy. Early diagnosis is challenging due to the frequent absence of positive cultures, making clinical history crucial. Prompt initiation of antituberculosis therapy often leads to rapid recovery. Clinicians should maintain a high index of suspicion in patients with persistent fever and other systemic symptoms, as early intervention significantly improves outcomes.
